# Coagulation activation, depletion of platelet granules and endothelial integrity in case of uraemia and haemodialysis treatment

**DOI:** 10.1186/1471-2369-14-72

**Published:** 2013-03-27

**Authors:** Marianne Schoorl, Margreet Schoorl, Menso J Nubé, Piet CM Bartels

**Affiliations:** 1 Department of Clinical Chemistry, Haematology & Immunology, Medical Center Alkmaar, Alkmaar, The Netherlands; 2 Department of Nephrology, VU Medical Centre, Amsterdam, The Netherlands

**Keywords:** Platelet granules depletion, Coagulation, Proendothelin, End-stage kidney disease, Renal failure

## Abstract

**Background:**

During haemodialysis (HD) treatment, increase of platelet (PLT) activation and induction of procoagulant activity is demonstrated. Although the role of the endothelium and its direct interaction with coagulation and homeostasis is known, it is not elucidated how PLT activation markers and activation of coagulation coincide with markers of endothelial integrity during HD treatment. In the present study uraemia and HD induced changes, with particular emphasis on PLT granules depletion, activation of coagulation and endothelial integrity were investigated.

**Methods:**

To detect depletion of PLT granules, peripheral blood slide smears were screened by light microscopy for qualitative evaluation of PLT granule containing cytoplasm, as indicated by its granules staining density. Activation of coagulation was investigated by establishement of thrombin-antithrombin (TAT) and fibrinogen concentrations. To evaluate endothelial integrity proendothelin (proET-1) plasma concentrations were established.

**Results:**

Results of our study demonstrate that proET-1 plasma concentrations were obviously increased in the subjects’ group with end-stage chronic kidney disease (CKD) and renal failure if compared with a group of apparently healthy subjects. The amount of depleted PLT granules was obviously increased in the subjects’ group with end-stage CKD if compared with the group with renal failure. Mean plasma concentrations of TAT and fibrinogen revealed results within the reference range.

**Conclusions:**

It is demonstrated that uraemia is associated with endothelial damage and aberrations in PLT granules morphology in subjects with HD treatment. We hypothesize that increased proET-1 concentrations reflect ongoing stress on endothelial cells amongst others due to uraemia. Biomarkers like proET-1 and aberrations in PLT granules morphology assist in the early detection of procoagulant activity of the endothelium.

## Background

Subjects with chronic kidney disease (CKD) are at risk of cardiovascular diseases and suffer from accelerated atherosclerosis [1,2]. Maintaining the functional integrity of the endothelium is important in prevention or delay of vascular diseases [3].

The vascular endothelium plays a pivotal role in the modulation of vascular tone, initiation of coagulation, fibrinolysis activity and release of inflammatory mediators [4].

The endothelium of subjects with CKD is continuously exposed to uraemic toxins. These toxins are classified in three groups: water-soluble compounds with low molecular weight, such as urea, middle molecular weight substances and protein-bound uraemic toxins [5]. Protein-bound uraemic toxins are poorly eliminated by haemodialysis (HD) treatment. Systemic exposure of the endothelium to uraemic toxins may lead to activation of the endothelial cells and to features associated with systemic inflammation like hypertension and atherosclerosis [6,7]. However, the mechanisms by which increased uraemia might influence activation of endothelial cells have not been elucidated.

Intact endothelium demonstrates anticoagulant activity [8]. An essential function of endothelium is to provide an anti-thrombotic surface which inhibits activation of the coagulation cascade [8]. Bacterial endotoxins or inflammatory cytokines, such as IL-1, and glycosylated proteins are able to activate endothelial cells [8]. Activated endothelium has procoagulant properties and promotes coagulation. Tissue factor originating from the endothelium plays an important role in the transformation of anticoagulant endothelium to procoagulant endothelium [8,9]. Agonists capable of inducing release of tissue factor include thrombin, endotoxins, cytokines, hypoxia, shear stress and oxidized lipoproteins. Shear stress and metabolic stimuli, in particular complement, granulocytes, platelets and free radicals, induce secretion of endothelin-1 (ET-1) and endothelial cell deterioration [10].

Subjects with end-stage CKD are on regular HD treatment for two or three times a week. Despite appropriate anticoagulation treatment, increase of platelet (PLT) activation and induction of procoagulant activity is demonstrated during HD treatment [11-14]. Thrombin is involved in the activation of PLTs, neutrophils and monocytes, and acts on endothelial cells in order to release vasoactive and inflammatory mediators [9].

Although the role of the endothelium and its direct interaction with coagulation and homeostasis is known, it is not elucidated how PLT activation markers and activation of coagulation interact with markers of endothelial integrity during HD treatment. In the current study we report on uraemia and HD induced changes, with particular emphasis on PLT granules depletion, activation of coagulation and endothelial integrity.

## Methods

### Patients

A group of 20 subjects with end-stage CKD (age 28–82 years) from the Haemodialysis unit of the Medical Center Alkmaar participated in the study. Patients were on regular HD treatment for at least 1 year (median 30 months, range 12–80 months). The etiology of chronic renal insufficiency was hypertensive nephrosclerosis (n = 8), diabetic nephropathy (n = 5), adult dominant polycystic kidney disease (n = 3), IgA nephropathy (n = 1), tubulo-interstitial nephritis (n = 1), chronic pyelonephritis (n = 1) and membranous nephropathy (n = 1). Criteria for exclusion were subjects with an age of < 18 years, a life expectancy < 3 months, active inflammation, thrombocytopenia, autoimmune disease or malignancy as well as supplementation of drugs interfering with PLT function or anticoagulation (immunosuppressive drugs, calcium antagonists, serotonin receptor antagonists, coumarin derivatives and salicylates). The study protocol was approved by the local Medical Ethical Committee (METC Noord-Holland, The Netherlands). Written informed consent was obtained from participants.

For the purpose of comparison and clinical interpretation with regard to the pathophysiological effects of uraemia, additional investigations were performed in a group of 20 subjects with renal insufficiency (aged 36–85 years, GFR < 80 ml/min).

A reference group of 20 subjects (laboratory technicians, aged 20–50 years), was selected in order to establish reference range intervals for parameters reflecting activation of coagulation and endothelial integrity.

### Blood sampling

During this study blood samples from the subjects’ group on regular HD treatment were taken from the fistula (t0) before the administration of LMWH.

For establishment of proendothelin-1 (proET-1) levels in plasma, blood samples were collected into K_2_EDTA-tubes (Vacutainer^®^, Becton Dickinson, Plymouth, UK). Sodium citrate tubes (0.109 Mol, Vacutainer^®^, Becton Dickinson, Plymouth, UK) were applicated for establishment of trombin-antitrombin (TAT) and fibrinogen plasma concentrations. Blood samples for determination of concentrations of proET-1, TAT and fibrinogen were centrifuged at 2-8°C for 20 minutes at 2500 g in order to separate plasma from the cellular fraction. Plasma aliquots were stored at -70°C until analysis.

### Analytical methods

#### Morphology of PLT granules

Peripheral blood slide smears were prepared in duplicate for evaluation of aberrations in the morphology of PLT granules. Slide smears were stained according to May-Grünwald-Giemsa methodology on a Sysmex SP-100 analyzer (Sysmex Corporation, Kobe, Japan). Slide smears were microscopically screened for qualitative evaluation of morphological PLT granules aberrations with application of a CellaVision™ DM96 analyzer (CellaVision AB, Lund, Sweden). As previously established, a staining density >75% of the PLT granules containing cytoplasm in >50% of PLTs was considered to be the lower limit of the reference range [15]. Depleted PLTs granules were defined as PLTs with a staining density amounting to <25% of the PLT granules containing cytoplasm. The upper limit of the reference interval for depleted PLT granules was determined at <20% of PLTs [15].

#### TAT, fibrinogen and proET-1 plasma concentration

TAT plasma concentrations were assayed with ELISA (Enzygnost^®^ TAT micro and Enzygnost^®^ F1 + 2 monoclonal, Siemens Healthcare Diagnostics Inc., Marburg, Germany). ProET-1 concentrations were established by means of a commercial LIA-kit (B.R.A.H.M.S. CT-proET-1, B.R.A.H.M.S. AG, Hennigsdorf, Germany). Fibrinogen concentrations were established on a ACL-TOP analyzer (Instrumentation Laboratory, Milan, Italy) in accordance with the Clauss method by adding excess of thrombin to diluted plasma in order to convert fibrinogen to fibrin (Instrumentation Laboratory, Milan, Italy).

### Statistical evaluation

Statistical evaluation of data was performed with application of SPSS software 14.0 for Windows. Statistical significance of deviations between mean values of the group of HD subjects and the group with renal failure and the reference group of laboratory technicians was established by applying the one-way analysis of variance (one-way ANOVA). A p-value < 0.05 was considered to be statistically significant. Correlation coefficients (r) were calculated and expressed as Pearson’s coefficients.

## Results

**Figure 1:**
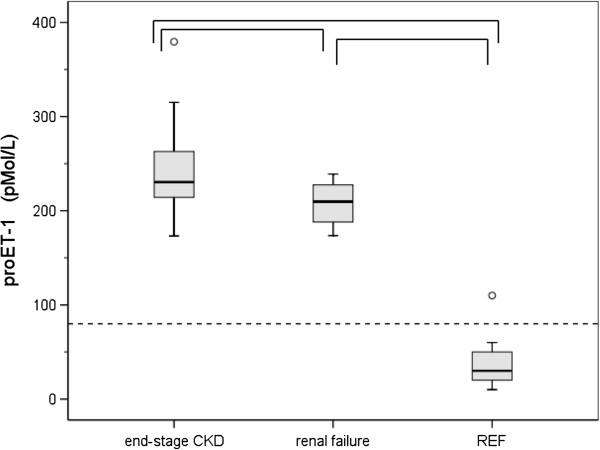
**Biomarker ProET-1 concentrations. **Box plots representing proET-1 concentrations established in subjects with end-stage CKD (n = 20) and renal failure (n = 20). For comparison, results for a group of 20 apparently healthy subjects (REF) are depicted. The box extends form the 25^th ^to the 75^th ^percentile. The line inside the box indicates the median value. Whiskers extend to the largest and smallest observed values within 1.5 box lengths. Outlying values corresponding with values between 1.5 and 3 times the box length are designated as (0). The horizontal dashed line indicates the upper level of the reference range for apparently healthy subjects. Statistically significant deviations between groups (p < 0.05) are indicated by ∏.

**Figure 2:**
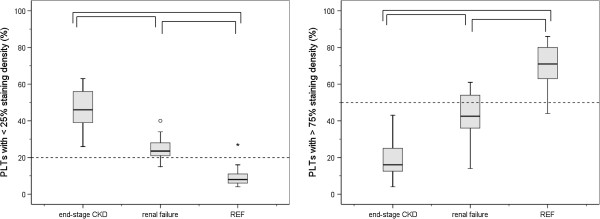
**PLT granules staining density. **Box plots representing the percentage of PLTs with <25% (left)) and >75% (right) staining intensity of the granules containing cytoplasm established in subjects with end-stage CKD (n = 20) and renal failure (n = 20). For comparison, results for a group of 20 apparently healthy subjects (REF) are depicted. The box extends form the 25^th ^to the 75^th^ percentile. The line inside the box indicates the median value. Whiskers extend to the largest and smallest observed values within 1.5 box lengths. Outlying and extreme values corresponding with values between 1.5 and 3 times the box length or > 3 times the box length, respectively, are designated as (0) and (*). The horizontal dashed lines indicate the upper (left) and lower (left) level of the reference range for apparently healthy subjects. Statistically significant deviations between groups (p < 0.05) are indicated by ∏.

**Figure 3:**
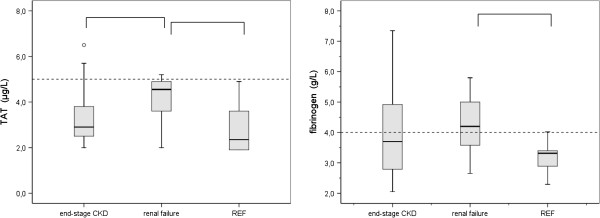
**Coagulation biomarkers TAT and fibrinogen. **Box plots representing TAT (left) and fibrinogen (right) plasma concentrations established in subjects with end-stage CKD (n = 20) and renal failure (n = 20). For comparison, results for a group of 20 apparently healthy subjects (REF) are depicted. The box extends form the 25^th^ to the 75^th ^percentile. The line inside the box indicates the median value. Whiskers extend to the largest and smallest observed values within 1.5 box lengths. Outlying values corresponding with values between 1.5 and 3 times the box length are designated as (0). The horizontal dashed line indicates the upper level of the reference range for apparently healthy subjects. Statistically significant deviations between groups (p < 0.05) are indicated by ∏.

Mean results for the groups of subjects with end-stage CKD and renal insufficiency together with the results of the reference group of laboratory technicians are depicted in the Figures 1, 2 and 3. Plasma concentrations of proET-1 are demonstrated in Figure 1. Results for parameters reflecting aberrations in the morphology of PLT granules are demonstrated in Figure 2. Results for parameters concerning activation of coagulation (TAT and Fibrinogen) are demonstrated in Figure 3.

### proET-1

ProET-1 concentrations in subjects with end-stage CKD (mean ± SD) amounted to 244 ± 47 pMol/L. One-way Anova statistics revealed a positive correlation regarding the reference group and the groups with renal insufficiency and end-stage CKD (p = 0.000). ProET-1 concentrations in the subjects with end-stage CKD (207 ± 23 pMol/L, p = 0.017) were significantly increased in comparison with subjects with renal insufficiency. ProET-1 results for both groups were obviously increased in comparison with the reference group of apparently healthy subjects (37 ± 22 pMol/L) (Figure 1). In 45% of the subjects with end-stage CKD proET-1 concentrations were above 250 pMol/L (Table 1).

**Table 1:** PLT granules staining density and coagulation activation parameters in end-stage CKD subjects with proET-1 concentrations of <250 and >250 pMol/L

	**End-stage CKD**		
	proET-1 <250 pMol/L (n = 11) mean ± SD	proET-1 >250 pMol/L (n = 9) mean (SD)	statistical significance
proET-1 (pMol/L)	210 ± 18	285 ± 40	p = 0.000
PLTs with <25% granules staining density (%)	33 ± 13	46 ± 8	p = 0.008
PLTs with >75% granules staining density (%)	34 ± 15	18 ± 10	p = 0.001
TAT (μg/L)	3.8 ± 1.0	3.8 ± 1.4	NS
Fibrinogen (g/L)	4.1 ± 1.3	3.9 ± 1.2	NS

Evaluation of proET-1, PLT granule staining density, TAT and fibrinogen (mean value with standard deviation) established in the group with end-stage CKD. Results of statistically significant evaluation are indicated as p-values.

NS = deviation not statistically significant.

### Morphology of PLT granules

In the group of reference subjects 70 ± 12% of the PLTs are established to reveal appropriate staining density of the PLT granules. In subjects with chronic HD treatment staining density of the granule containing PLT cytoplasm decreased to a minimum score (Figure 2). Only 19 ± 10% of the PLTs yielded > 75% PLT granules staining density (p = 0.000). Subjects with renal failure showed also a marked decrease of the PLTs with appropriate staining density of the PLT granules (43 ± 12%, p = 0.000).

PLTs with a staining density amounting to <25% of the PLT granules containing cytoplasm are classified as depleted. In the reference subjects’ group only 9 ± 6% of the PLTs reveal depleted PLT granules (p = 0.000) (Figure 2). In the subjects’ group with end-stage CKD 46 ± 11% of the PLTs reveal depleted granules staining. In the subjects’ group with renal failure 25 ± 6% of the PLTs reveal depleted granules staining.

### TAT, fibrinogen

TAT plasma concentrations and fibrinogen concentrations in the group with end-stage CKD and renal failure are demonstrated in Figure 3. One-way Anova statistics revealed a positive correlation regarding the reference group and the groups with renal insufficiency and end-stage CKD (p = 0.010). Mean results for TAT and fibrinogen concentrations in the group with end-stage CKD were within the reference range and did not statistically differ from the results of the reference group. However, in the group with end-stage CKD increased TAT or fibrinogen concentrations were established in respectively 15% and 40% of the subjects.

Statistically significant increases for fibrinogen and TAT concentrations were established in the subjects with renal failure in comparison with the reference group (p = 0.000), whereas only TAT results differ significantly from the results of the group with end-stage CKD (p = 0.014).

### Correlation between proET-1 and modifications in PLT morphology or markers indicating activation of coagulation

**Figure 4:**
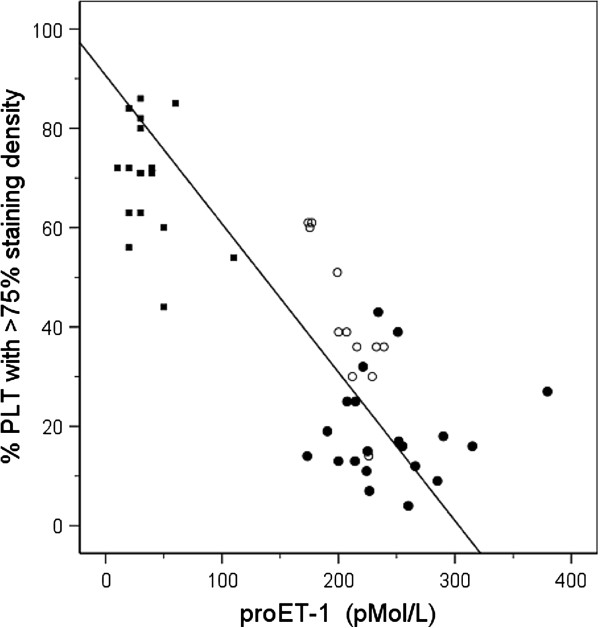
**Correlation between PLT granules staining density and biomarker proET-1.** Relationship between results concerning PLT granules staining density and proET-1 concentrations in the groups with end-stage kidney disease (●), renal failure (o) and apparently healthy subjects (■) respectively.

Establishment of the interdependence of proET-1 results with additional parameters reflecting aberrations in the morphology of PLT granules reveal a negative correlation with PLTs with appropriate PLT granules staining density r = -0.84, p = 0.000 (Figure 4).

For results concerning activation of coagulation a similar correlation is detected (Table 2). A statistically significant positive correlation to increased values for proET-1 with TAT and fibrinogen has been established in the group of reference subjects and the subjects groups with renal failure and end-stage CKD of r = 0.34 (p = 0.016) and r = 0.31 (p = 0.028) respectively (Table 2).

**Table 2:** Pearson correlation coefficients (r) and statistical significance (p) between biomarker proET-1, PLT granules staining density and biomarkers indicating activation of coagulation

	**Pearson correlation (r)**	**Statistifical significance**
proET-1 and PLTs with <25% granules staining density	0.79	p = 0.000
proET-1 and PLTs with >75% granules staining density	-0.84	p = 0.000
proET-1 and TAT	0.34	p = 0.016
proET-1 and Fibrinogen	0.31	p = 0.028

Nine patients (45%) of the subjects' group with end-stage CKD yielded proET-1 concentrations exceeding 250 pMol/L (Table 1). Results for aberrations in the morphology of PLT granules in the subjects’ group with proET-1 concentrations exceeding 250 pMol/L demonstrated statistically significant deviations if compared with the subjects’ group with proET-1 concentrations below 250 pMol/L, whereas markers for activation of coagulation did not differ (Table 1).

## Discussion

ProET-1 plasma concentrations in subjects with end-stage CKD and renal failure were investigated for a possible association between reduced endothelial integrity and aberrations in the morphology of PLT granules or markers indicating activation of coagulation.

The *in vivo* inactive biomarker proET-1 reflects the level of the bioactive peptide ET-1. ProET-1 is the precursor of ET-1, which reveals stability *ex vivo*[16]. Results of the study demonstrate that ProET-1 plasma concentrations are obviously increased in the subjects’ group with end-stage CKD and renal failure if compared with a group of apparently healthy subjects. Results are in accordance with the findings of other authors, who demonstrated increasing endothelin plasma concentrations with progression of renal failure [17,18]. Altered expression of microcirculation parameters with age is a commonly occurring phenomenon. In the age of 25 till 65 years concentrations of proET-1 increase by 20% [16]. However, on the basis of age related shifts, the increase in proET-1 concentrations in the groups with end-stage CKD and renal failure are obviously higher than expected. ProET-1 plasma concentrations in the subjects’ group with end-stage CKD demonstrate a statistically significant increase if compared with the subjects’ group with renal insufficiency. Endothelial injury is considered to initiate increased secretion of ET-1 and to effectuate vasoconstriction, increased intraglomerular pressure, and decreased glomerular filtration [17]. ET-1 concentrations are demonstrated to correlate with blood pressure, suggesting that ET-1 may contribute to hypertension [18]. Reduced endothelial integrity is considered to be associated with increased incidence of cardiovascular disease [17]. Together with inflammation, hyperhomocysteinaemia and anaemia, cardiovascular disease yields an additional risk factor in subjects with end-stage CKD [19].

Moreover, results of our study demonstrate that the amount of depleted PLT granules is obviously increased in the subjects’ group with end-stage CKD if compared with the group with renal failure. Subjects with end-stage CKD are on regular HD-treatment. Pathophysiological mechanisms inducing activation of coagulation are based on Virchow’s triad including modifications in vessel wall, blood flow and composition of blood components [20-22]. During HD treatment, blood constituents interact with the foreign surfaces within the extracorporeal circuit (ECC), including the wall of blood lines, the artificial dialyzer membrane and mechanical forces of the roller pump. Within the ECC endothelium is lacking and activation of PLTs and biomarkers inducing activation of coagulation are released by mechanical triggers [23]. In order to prevent clotting in the ECC during HD, a bolus of low molecular weight heparin or unfractionated heparin is supplied at the start of each HD session. Despite appropriate anticoagulation treatment, the rather unphysiological conditions within the ECC, amplified by pre-dialysis increased uraemia related factors, induce PLT activation. Increase of PLT activation and procoagulant activity is demonstrated in HD patients [24,25]. PLTs are activated within the ECC, as detected by increase in the expression of CD62p and release of β-tromboglobulin (β-TG) within the ECC [23]. PLT activation is associated with exposure of phosphatidylserine on the PLT exterior. Platelet factor-4 and β-TG are released from PLTs as a result of a defect in their granules membrane mainly as a consequence of the blood-membrane contact during HD and return only slowly postdialytic to control values [26].

In this study mean concentrations of TAT and fibrinogen reveal results within the reference range. However, in the group with end-stage CKD increased TAT or fibrinogen concentrations are established in respectively 15% and 40% of the subjects, indicating activation of the coagulation pathway or an acute phase response already before the beginning of a new HD session. The inner negatively charged wall of the ECC induces activation of FXII and subsequently the intrinsic coagulation pathway. Increased concentrations of TAT as well as prothrombin fragment 1 + 2 treatment during HD indicate that thrombin is generated [12-14]. Additionally, it has been demonstrated that thrombin is involved in the activation process of PLTs, neutrophils, and monocytes, and acts on endothelium in order to release a variety of vasoactive and inflammatory mediators [9].

With respect to different biomarkers of activation of PLTs and the coagulation pathway dissimilar results could be obtained. Although activation of PLTs and the coagulation pathway is present, concentrations of activation and release products staying within the reference values could also be detected as a result of different release times, presence of neutralizing agents and removal by the dialyzer membrane [11,14].

Results of our study demonstrate that uraemia is associated with endothelial damage and aberrations in the staining density of PLT granules in subjects with CKD. Uraemic toxins, especially protein-bound toxins, are likely pathogenic agents inducing endothelial damage in CKD [27]. In case of renal failure, endothelial damage and cardiovascular complications like hypertension are closely linked [28,29]. Concerning interpretation of our experimental data, we hypothesize that increased proET-1 concentrations reflect ongoing stress on the endothelium amongst others due to uraemia. Biomarkers like proET-1 and aberrations in the staining density of PLT granules contribute in the early detection of procoagulant activity of the endothelium. Therefore, the level of proET-1 concentration and the amount of depleted PLT granules will add an important link to the degree of endothelial integrity and the severity of CKD.

## Conclusions

In this study it has been demonstrated that uraemia is associated with endothelial damage and aberrations in appropriate staining density of PLT granules in subjects with HD treatment. In subjects with end-stage CKD deterioration of integrity of the endothelium and depletion of PLT granules are aggravated, because of the frequent PLT activation in the ECC during HD treatment.

## Abbreviations

CKD: Chronic Kidney Disease; ET-1: Endothelin-1; HD: Haemodialysis; PLT: Platelet; TAT: Trombin-antitrombin.

## Competing interest

The authors have no financial or other competing interests.

## Authors’ contributions

MiS participated in the design of the study, analysis of laboratory parameters for platelet granules staining density, activation of coagulation and endothelial integrity and data interpretation and has written the manuscript. MgS participated in the analysis of laboratory parameters for platelet granules staining density and activation of coagulation and has read and approved the manuscript. MN participated in the design of the study, provided intellectual content of haemodialysis and importance of the work, helped with a draft version of the manuscript and has read and approved the manuscript. PB participated in the design of the study and design and data interpretation, provided intellectual content of platelet granules staining density, activation of coagulation and endothelial integrity and importance of the work, has critically revised the draft versions of the manuscript and gave final approval for the current manuscript.

## Pre-publication history

The pre-publication history for this paper can be accessed here:

http://www.biomedcentral.com/1471-2369/14/72/prepub
